# Combined Transcriptomic and Histological Profiling Uncover Hepatic Regulatory Hierarchy of Triploid *Oncorhynchus mykiss* Under Interactive Salinity, Temperature and Body Weight Regimes

**DOI:** 10.3390/biology15181646

**Published:** 2026-09-17

**Authors:** Yanming Sui, Yan Ji, Haopeng Hu, Yuanhao Ren, Bo Qin, Na Ying, Tingting Lin, Siping Li, Hanfeng Zheng, Lei Li

**Affiliations:** 1 Key Laboratory of East China Sea Fishery Resources Exploitation, Ministry of Agriculture and Rural Affairs, East China Sea Fisheries Research Institute, Chinese Academy of Fishery Sciences, Shanghai 200090, China; suiyanming@ycit.edu.cn (Y.S.); hu15231265111@163.com (H.H.); renyh@ecsf.ac.cn (Y.R.); dhsqb1026@126.com (B.Q.); xyz6622@126.com (N.Y.); lintt@ecsf.ac.cn (T.L.); lisiping@ecsf.ac.cn (S.L.); 2 Jiangsu Key Laboratory for Exploration and Utilization of Marine Wetland Biological Resources, Yancheng Institute of Technology, Yancheng 224051, China; 13182602706@163.com; 3 Jiangsu (Marine) Salmon and Trout Science and Technology Innovation Center, Yellow Sea Laboratory for Blue Seed Industry, Yancheng 224000, China

**Keywords:** rainbow trout, liver, orthogonal design, alternative splicing, WGCNA, salinity acclimation

## Abstract

Salinity, temperature and body weight dominate seawater acclimation in rainbow trout (*Oncorhynchus mykiss*), yet few studies simultaneously explore their main effects and potential correlative interactive patterns in hepatic responses. A 60-day L_9_ (3^3^) orthogonal trial was performed on triploid rainbow trout with three gradients of body weight, temperature and salinity. Hepatic transcriptomics revealed that salinity drove global transcriptional remodeling and high salinity induced far fewer DEGs than medium salinity. WGCNA screened a salinity-positive blue module (*r* = 0.408, *p* = 0.0346), while alternative splicing confirmed extensive salinity-dependent post-transcriptional regulation. Semi-quantitative histology showed that 20 °C was associated with more pronounced salinity-caused hepatocellular vacuolation and karyopyknosis in the orthogonal test. Survival statistics indicated that salinity was the only factor with significant main effects (*p* < 0.05), and the 500 g-10 °C-10 ppt group obtained the highest survival. This multi-omics and histological dataset reveals a suggestive regulatory hierarchy-like pattern: salinity acts as the primary driver, temperature serves as a synergistic amplifier, and body weight plays a minor modulatory role. These findings provide a theoretical basis for developing size-specific salinity acclimation protocols in commercial triploid rainbow trout farming.

## Simple Summary

Seawater domestication is a promising way to expand the aquaculture production of triploid rainbow trout (*Oncorhynchus mykiss*), yet how body weight, temperature and salinity jointly shape hepatic adaptive responses remains poorly understood. Using an L_9_ orthogonal trial combined with liver histopathology and transcriptome sequencing, our study shows that salinity acts as the primary driver of survival and hepatic molecular reprogramming, while temperature is associated with salinity-triggered liver injury and body weight exerts relatively minor modulatory effects. Medium salinity induced far more transcriptomic and alternative splicing changes than extreme high salinity. Our results provide practical references for the size-dependent brackish water domestication of farmed triploid rainbow trout. We also clarify several experimental limitations of the orthogonal design, which should be considered in future multi-factor stress studies in teleost fish.

## 1. Introduction

Rainbow trout (*Oncorhynchus mykiss* (Walbaum, 1792)) is a typical coldwater fish whose growth, survival and physiological homeostasis rely on low-temperature habitats. Global warming has triggered persistent temperature rises across natural aquatic habitats, and the co-occurrence of elevated temperatures and unstable salinity greatly limits its aquaculture production capacity, which makes the optimization of seawater domestication protocols an urgent demand for aquaculture production. As the most widely farmed cold salmonid worldwide, rainbow trout possesses excellent edible quality and high economic value, with a global aquaculture output of 960,000 tons in 2020 [1]. Introduced to China in the late 1950s, this species has been commercialized in large-scale aquaculture industries in highland coldwater regions including Gansu, Qinghai and Xinjiang after over six decades of breeding and promotion [2]. Nevertheless, the sustained rising domestic demand for high-quality rainbow trout is confronted with severe constraints on inland freshwater aquaculture expansion: cold alpine freshwater resources are scarce, while increasingly stringent ecological control policies further limit the scale of traditional land-based farming. China’s freshwater rainbow trout output only reached 37,000 tons in 2022, against an annual domestic market demand of 120,000 tons [1,2], creating a prominent supply–demand gap. To address insufficient production capacity, domestic aquaculture practitioners have gradually explored brackish water domestication and offshore cage culture modes. Offshore cage trials conducted in Ganyu, Jiangsu, recorded a survival rate of 72% and an average individual weight gain of 650 g after 5 months of seawater domestication, verifying the operability of marine farming [3]. Unlike stable, controllable inland freshwater ponds, offshore aquaculture waters exhibit drastic environmental fluctuations driven by seasonal shifts, rainfall runoff and tidal cycles: surface water temperature can surge by 5–7 °C within a single day during summer heatwaves, rainfall runoff may reduce local salinity by 6–10 ppt in a short period, and seasonal alternation triggers months-long continuous deviations in thermal and saline parameters. Synchronous variations in temperature and salinity generate synergistic or antagonistic compound stress effects on fish physiology [4]. At present, prominent gaps exist in both industrial practice and academic research: the optimal temperature–salinity combinations matching rainbow trout of different body weights remain undefined, and systematic analyses on adaptive metabolic regulation under multi-environment coupling stress are lacking, which severely hinders the standardization of domestication techniques. The regulatory patterns of fish metabolism under the combined effects of temperature, salinity and body weight remain unclear. Gill, kidney and intestine represent the primary osmoregulatory organs in teleost fish. The liver mainly suffers secondary metabolic disturbances triggered by osmotic imbalance and serves as a critical target tissue for analyzing downstream physiological consequences under osmotic stress responses, yet systematic research on the hepatic responses of rainbow trout under the interactive stress of the three factors is still sparse.

The liver, though not a primary osmoregulatory organ, undergoes profound secondary metabolic perturbations triggered by osmotic imbalance and acts as a key target tissue reflecting the downstream physiological consequences of osmotic stress, energy metabolism and antioxidant defense in fish, representing the core tissue in the evaluation of physiological responses to multiple environmental stressors. Osmoregulation constitutes the key physiological pathway for euryhaline fish to adapt to salinity changes, which is jointly regulated by species genetics, developmental stage, water temperature, salinity and body weight, with distinct interspecific regulatory discrepancies. Existing studies have proven the significant interactive effects of temperature and salinity on fish salinity tolerance [5,6]: for Nile tilapia, the 96 h median lethal salinity drops from 26.2 to 16.6 °C within the temperature range of 25–31 °C, which indicates strong thermal–salinity crosstalk [4]. Combined high temperature and low salinity significantly alter hepatic SOD and CAT activities as well as MDA levels in Japanese flounder, which confirms the significant interactive effects of temperature and salinity on hepatic oxidative stress indicators [7]. The salinity tolerance threshold of brown trout is directly modulated by water temperature [8], while the seawater acclimation efficiency of European sea bass is co-determined by temperature and photoperiod [7]. Current research on rainbow trout mostly adopts single-factor experimental designs. Maryoung et al. characterized differential gene expression in the liver, gill and olfactory rosettes of coho salmon after salinity acclimation [9]. Yang et al. characterized hepatic transcriptional alterations triggered by isolated high-temperature treatment [10]. A small number of recent studies only designated dual temperature–salt groups without incorporating fish body weight as a core variable [11,12]. To date, no published research has integrated body weight, temperature and salinity into one experimental system via orthogonal design to simultaneously resolve their individual main effects. By contrast, orthogonal array designs have been successfully applied in multi-factor aquaculture studies, including in the evaluation of salinity–alkalinity combined toxicity in fish [13]. Comprehensive, systematic interpretations of how multi-factor coupling modulates hepatic osmotic adaptation in rainbow trout are absent in the existing literature; there remains a critical research gap with regard to integrated histological and multi-omics evidence. Transcriptomics combined with histopathology provides a reliable analytical tool for evaluating multi-layer hepatic responses under compound stress, overcoming the limitations of single-factor experiments.

RNA-seq is a high-throughput omics technology for profiling genome-wide transcriptional reprogramming under environmental stress, which facilitates the high-throughput screening of DEGs, genome-wide detection of alternative splicing (AS) events, and construction of gene co-expression networks, and it has been widely applied in stress-related research on aquaculture fish [14]. This technique has been adopted maturely in salinity adaptation studies of euryhaline species: a liver transcriptome analysis of large yellow croaker under low salinity clarified core lipid metabolism and osmoregulatory pathways [15]. Transcriptomic detection of domestic rainbow trout strain “Shuike No. 1” under dual thermal–salinity stress verified that endoplasmic reticulum protein processing serves as the central stress pathway [11]. Nevertheless, transcriptomic data alone cannot intuitively reflect real tissue damage status. Hematoxylin–eosin (HE) histopathological observation acts as a complementary method that can directly visualize hepatocellular vacuolation, pyknosis and inflammatory infiltration, and it has been widely applied in fish hepatic injury assessment [16,17]. Integrating transcriptomic molecular data and hepatic histological morphology enables the comprehensive restoration of fish adaptive status under compound osmotic stress at the molecular and tissue levels, compensating for the drawbacks of single-omics analysis.

In this study, triploid rainbow trout were selected as experimental subjects. Three core factors—water temperature, salinity and fish body weight—were set, and an L_9_ (3^3^) orthogonal experimental design was applied to explore main-factor effects and potential correlative interactive trends among temperature, salinity and body size classes [2]. Compared with conventional single- or two-factor experimental schemes, the orthogonal design better simulates the synchronous fluctuation of multiple environmental variables in actual offshore aquaculture and is rarely reported in salmonid liver-related research. Hepatic RNA sequencing and HE histological observation were performed across all nine treatment groups. By integrating histological and transcriptomic data, this study aims to clarify hepatic adaptive regulatory mechanisms under multi-factor coupling stress: our objectives are to (1) characterize hepatic morphological injury features under distinct temperature–salinity combinations via HE staining [18]; (2) screen core genes and enriched pathways regulating energy supply, antioxidant capacity and endoplasmic reticulum stress [19]; (3) illustrate the independent and interactive regulatory patterns of the three factors on hepatic transcriptional profiles [20]; and (4) screen hub marker genes to evaluate the salinity tolerance of large-bodied rainbow trout [21]. This research constructs an integrated “histopathology + transcriptomics” analytical framework, filling the research gap regarding the hepatic adaptive mechanisms of triploid trout exposed to multi-factor compound stress [2]. The present results provide histological and molecular theoretical support for establishing size-specific seawater domestication protocols and promoting sustainable brackish and offshore rainbow trout aquaculture in China [2,22].

## 2. Materials and Methods

### 2.1. Animal Ethics Statement

For all animal experiments in this study, we strictly followed the guidelines for the care and use of laboratory animals formulated by the Animal Ethics Committee of the East China Sea Fisheries Research Institute, Chinese Academy of Fishery Sciences. All experimental protocols were reviewed and approved by the Animal Ethics Committee (Approval No. 2024-22). During sampling, fish were anesthetized with 40 mg/L MS-222 to minimize pain and suffering. All operations were carried out in accordance with national standards for aquatic animal welfare in China.

### 2.2. Experimental Fish and Orthogonal Salinity Acclimation Experiment

#### 2.2.1. Pre-Experiment Acclimation

Triploid rainbow trout (*O. mykiss*) were induced by hydrostatic pressure to block second polar body extrusion during fertilization. Before formal salinity domestication, healthy fish of three weight specifications were raised in indoor circulating tanks for a 15-day acclimation period. The culture environment was set to a 12 h light/12 h dark photoperiod, with water temperature stabilized at 15 ± 1 °C, dissolved oxygen maintained at 7.8–10.0 mg/L, and pH 7.1–7.5. Fish were fed commercial compound feed twice daily at 2% body weight. Individuals with intact body surface and uniform initial weight (100 ± 10 g, 250 ± 10 g, 500 ± 20 g) were screened and fasted for 24 h prior to the start of formal trials.

#### 2.2.2. Orthogonal Experimental Design

A three-factor, three-level L_9_ orthogonal experimental design was adopted to evaluate main-factor effects and observe potential correlative trends in body weight (Factor A), water temperature (Factor B) and salinity (Factor C). The setting of each factor level is displayed in Table 1, and the complete grouping scheme of nine treatments is listed in Table 2. Each treatment group was equipped with two replicate 600 L tanks, and 30 triploid rainbow trout were raised in each tank.

The three gradient levels for body weight (100 g, 250 g, 500 g), temperature (10 °C, 15 °C, 20 °C) and salinity (10 ppt, 20 ppt, 30 ppt) were selected based on published reports describing seawater acclimation performance of triploid rainbow trout. After the 15-day pre-acclimation at 15 ± 1 °C, temperature and salinity were ramped simultaneously and gradually over 7 consecutive days until reaching target experimental levels before the formal 60-day trial. During pre-acclimation, fish of three weight classes were cultured in separate tanks to avoid inter-individual competition and density-related confounding effects. A unified 24 h fasting was performed for all groups prior to the onset of formal treatments. We acknowledge that longer fasting duration would benefit gut clearance for large-bodied fish, yet gut content quantification was not conducted in this study.

Each orthogonal treatment contained two independent replicate 600 L tanks, with 30 triploid rainbow trout stocked per tank. Primary outcome variables measured in this trial included survival rate, weight gain performance, hepatic histopathological injury, and hepatic transcriptomic responses. Plasma osmolality, cortisol and feed conversion ratio were not assayed in the present experiment. Tissue sampling was carried out at the endpoint of the 60-day formal culture trial. For each replicate tank, 12 fish were anesthetized and dissected: three individuals for transcriptome sampling, six individuals for histological embedding, and remaining fish for survival and growth statistics.

It is acknowledged that the L_9_ orthogonal array is primarily designed for estimating main effects and that effect aliasing precludes robust independent estimation of all pair-wise interactions. Therefore, any observed interactive-like patterns reported in this study should be interpreted as correlative trends rather than statistically confirmed causal interactions. Formal testing of two-factor interactions will require full-factorial designs in future work.

#### 2.2.3. Daily Culture Management

Commercial artificial sea salt was used to adjust salinity, which was stabilized within ±0.5 ppt of target values after the 7-day ramping phase and maintained throughout the subsequent 60-day trial. Circulating heating/refrigeration equipment kept temperature fluctuation ≤±0.2 °C. Fish were fed daily at 09:00 and 17:00 to apparent satiation (ad libitum feeding) during formal trial, whereas a fixed feeding rate of 2% body weight per day was adopted during pre-acclimation. Residual feed and feces were siphoned off daily. Ammonia-N and nitrite-N were monitored twice per week and maintained below toxic thresholds. Water temperature, salinity, dissolved oxygen and pH were recorded daily, and photoperiod remained consistent with pre-acclimation. Liver tissues were collected upon termination of the 60-day experiment.

#### 2.2.4. Tissue Sampling

After the 60-day culture period, 12 fish per tank were anesthetized and dissected for liver sampling. Remaining fish were counted and weighed to calculate survival and weight gain rate.

Transcriptome sampling: Three fish per group, liver tissue snap-frozen in liquid nitrogen and stored at −80 °C for RNA extraction;

Histological sampling: Six fish per group, hepatic tissues fixed in 4% paraformaldehyde at 4 °C for 24 h, then transferred to 70% ethanol for long-term preservation before embedding.

Notably, due to the orthogonal layout with multiple varying factors between single groups, direct pair-wise comparison of individual treatments was not performed. All phenotypic and molecular data were analyzed via main-effect grouping and two-factor interaction models to eliminate confounding interference.

### 2.3. Hepatic Histopathological Observation

#### 2.3.1. Paraffin Embedding and HE Staining

Fixed liver samples underwent gradient ethanol dehydration and xylene clearing followed by paraffin embedding. A rotary microtome was used to prepare continuous 4–5 μm slices. Sections were dewaxed, rehydrated and stained with standard hematoxylin–eosin (HE) procedures: hematoxylin nuclear staining, hydrochloric acid alcohol differentiation, ammonia water bluing, and eosin cytoplasmic counterstain, then dehydrated, made transparent and sealed with neutral balsam.

#### 2.3.2. Microscopic Observation

Slices were photographed under an Olympus BX53 optical microscope (Olympus, Tokyo, Japan) at 100× and 400× magnification. Six non-overlapping random visual fields were captured per sample for the observation of hepatocellular vacuolation and nuclear pyknosis. A blind semi-quantitative scoring system conducted by two independent observers was adopted to evaluate hepatic lesion severity: 0 = intact normal hepatic tissue with no pathological lesions; 1 = mild injury, scattered lesions occupying < 25% of the visual field; 2 = moderate injury, lesions covering 25–50% of the visual field; 3 = severe injury, diffuse lesions occupying > 50% of the visual field. Corresponding scoring values were recorded for statistical analysis.

### 2.4. RNA Extraction, Quality Detection and Library Construction

Total RNA was extracted from frozen liver tissue using TRIzol reagent (Invitrogen, Carlsbad, CA, USA), and DNase I was added to remove genomic DNA contamination. RNA purity was measured by NanoDrop 2000 spectrophotometer (Thermo Fisher, Waltham, MA, USA), with OD_260_/OD_280_ controlled at 1.8–2.2. RNA concentration was quantified via Qubit 2.0, and integrity was detected using Agilent 5300 Bioanalyzer (Agilent, Santa Clara, CA, USA). Only samples with RIN ≥ 7.0 were retained for library construction, with three biological replicates per orthogonal group [23].

Three micrograms of qualified total RNA were used for strand-specific PE150 libraries. Oligo(dT) magnetic beads enriched polyA mRNA, followed by random fragmentation, first-/second-strand cDNA synthesis, end repair, A-tailing and adapter ligation. Purified fragments (200–300 bp) were amplified by PCR, and qualified libraries were sequenced on Illumina NovaSeq X Plus platform (Illumina, San Diego, CA, USA) with paired-end 150 bp mode.

### 2.5. Raw Data Filtering, Genome Alignment and Transcript Annotation

All bioinformatic software and R packages used for RNA-seq analysis were as follows: fastp v0.23.4, HISAT2 v2.2.1, StringTie v2.2.0, rMATS v4.0.1, DESeq2, clusterProfiler v4.12.0, ggplot2 and pheatmap under R v4.2.1. Subsequent raw data processing, genome alignment and functional annotation were performed with the above uniform versions.

Raw sequencing reads were processed with fastp to strip adapters and filter low-quality reads (sliding window = 4:15, min length ≥ 50 bp). Clean reads were mapped to rainbow trout reference genome (GCF_013265735.2) via HISAT2. StringTie was adopted for transcript assembly and read count statistics; gene expression was normalized to FPKM and TPM simultaneously. All transcripts were annotated against Nr, Swiss-Prot, GO, KEGG, and Pfam databases with E value < 1 × 10^−5^.

### 2.6. Differential Alternative Splicing (DAS) Identification

rMATS was used to identify five categories of alternative splicing events: exon skipping (ES), intron retention (IR), mutually exclusive exon (MXE), alternative 5′ splice site (A5SS), and alternative 3′ splice site. Events with FDR < 0.05 were identified as DAS events, and isoform inclusion levels were calculated to compare splicing patterns among main-effect subgroups.

### 2.7. DEG Screening and Multi-Dimensional Enrichment Analysis

Differentially expressed genes (DEGs) were screened using the DESeq2 R package, thresholds |log_2_FC| ≥ 1, adjusted *p* (FDR) < 0.05. GO and KEGG enrichment of DEGs and DAS-related genes were conducted with clusterProfiler. Gene Set Enrichment Analysis (GSEA) was further performed to excavate subtle pathway changes, with *p* < 0.05 as the significance cutoff.

### 2.8. Sample Clustering and PCA Analysis

Principal component analysis (PCA) and hierarchical clustering heatmaps based on all gene FPKM values were drawn via pheatmap and prcomp functions in R v4.2.1 to evaluate the repeatability of biological replicates and overall transcriptome differentiation among groups.

### 2.9. Weighted Gene Co-Expression Network Analysis (WGCNA) Analytical Procedures

Weighted gene co-expression network analysis (WGCNA) was performed using all expressed genes from 27 hepatic transcriptome samples (*n* = 3 biological replicates per orthogonal group). Soft-threshold power β = 7 was chosen to satisfy scale-free topology standard (R^2^ ≥ 0.85) for subsequent co-expression network construction. Module–trait correlation analysis was conducted against five variables: salinity gradient, temperature gradient, body weight class, survival rate and weight gain rate. Modules significantly correlated with phenotypic traits were screened, and hub genes inside key modules were extracted as potential salinity tolerance biomarkers.

### 2.10. Statistical Analysis

Statistical testing was exclusively conducted on growth performance phenotypes (survival rate, weight gain); transcriptomic and histological results were analyzed by qualitative description and omics bioinformatic methods without variance analysis. Survival rate and weight gain data were expressed as mean ± standard deviation (mean ± SD).

Orthogonal range analysis was adopted for preliminary trend observation of growth performance; significance evaluation relied on three-way ANOVA (Response~Temperature + Salinity + Temperature × Salinity). Body weight was not included as a factor in this ANOVA model because the L_9_ orthogonal array does not provide sufficient degrees of freedom to simultaneously estimate the main effects of all three factors and their two-way interactions within a single full-factorial ANOVA framework. Instead, body weight effects were examined separately using one-way ANOVA after grouping samples by weight class (small, medium, and large), as described in the main-effect grouping rule above. It is acknowledged that this approach does not allow formal testing of interactions involving body weight, and the related findings were interpreted with appropriate caution. Interaction term *p* < 0.05 was defined as statistically significant.

Main-effect grouping rule:

Salinity: Low (10 ppt, Groups 1/5/9), medium (20 ppt, Groups 2/6/7), and high (30 ppt, Groups 3/4/8);

Temperature: Low (10 °C, Groups 1/6/8), medium (15 °C, Groups 2/9), and high (20 °C);

Body weight: Small (100 g, Groups 1/2/3), medium (250 g, Groups 4/5/6), and large (500 g, Groups 7/8/9).

One-way ANOVA combined with Tukey’s multiple comparison test was applied for growth-related main group comparisons; *p* < 0.05 represented significant difference.

All transcriptome-related visualizations were generated using the ggplot2 package in R v4.2.1. Statistical analyses of growth phenotypes and omics data were performed via SPSSAU 26.0 and R 4.2.1.

All RNA quantification, sequencing processing, differential gene screening, functional enrichment and visualization were performed using unified bioinformatic tools and R packages [18,24,25,26,27,28,29,30,31,32,33]. Standard biostatistical analysis was adopted for phenotypic data [31].

## 3. Results

### 3.1. Hepatic Histopathological Lesions Under Nine Orthogonal Culture Treatments

**Figure 1:**
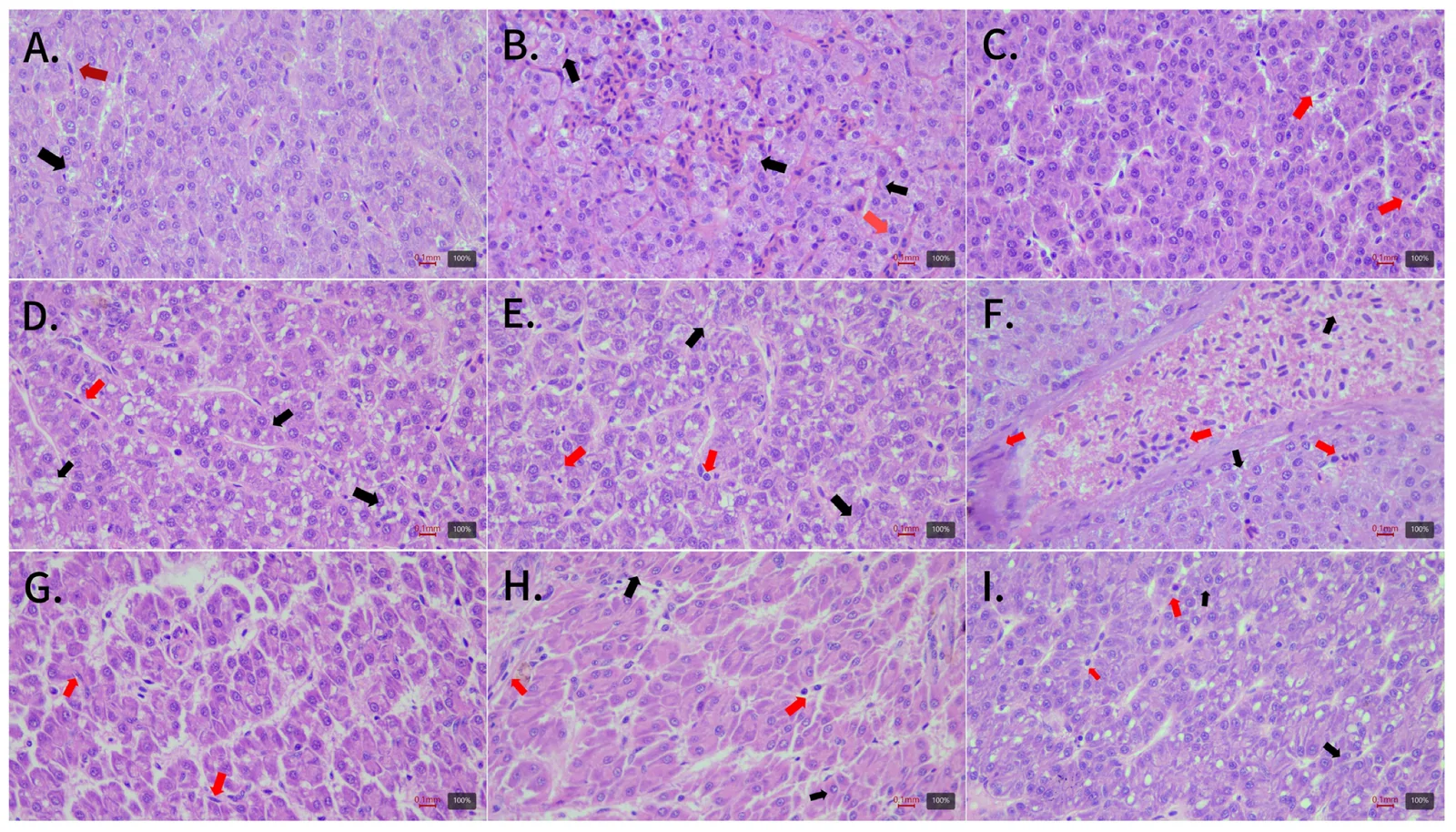
Representative HE-stained hepatic tissue micrographs of triploid rainbow trout under nine orthogonal culture treatments (400× magnification, scale bar = 0.1 mm). Subgraphs (**A**–**I**) correspond to nine orthogonal groups, respectively: (**A**) 100 g-10 °C-10 ppt, (**B**) 100 g-15 °C-20 ppt, (**C**) 100 g-20 °C-30 ppt, (**D**) 250 g-15 °C-30 ppt, (**E**) 250 g-20 °C-10 ppt, (**F**) 250 g-10 °C-20 ppt, (**G**) 500 g-20 °C-20 ppt, (**H**) 500 g-10 °C-30 ppt, and (**I**) 500 g-15 °C-10 ppt. Black arrows: hepatocellular vacuolation; red arrows: karyopyknosis.

Representative 400× HE-stained hepatic sections from nine experimental groups are assembled into a 3 × 3 grid (Figure 1). Distinct hepatic tissue morphological differences were observed across temperature–salinity combined treatments. Group 1, Group 7 and Group 9 exhibited lesion scores of 0–0.5, showing neatly arranged hepatic cords and homogeneous cytoplasm, with only sporadic cellular changes; the overall liver tissue architecture remained nearly intact. Moderate pathological alterations (lesion scores 1.0–1.8) were observed in Group 4, Group 5 and Group 8, where scattered hepatocellular vacuolation (Figure 1D) and sporadic karyopyknosis (Figure 1H) could be detected, while hepatic cord structure was largely preserved. Group 2, Group 3 and Group 6 obtained higher lesion scores ranging from 2.5–3.0, showing diffuse cytoplasmic vacuolation and massive karyopyknosis, which caused severe disruption of regular hepatic cord arrangement and reflected substantial hepatic structural damage. Consistent with transcriptome enrichment results of apoptosis and oxidative stress pathways [31], our observations imply that high temperatures may aggravate hepatic tissue injury under high-salinity conditions. Semi-quantitative scoring of hepatic lesions adopted the mature evaluation system established for salinity-stressed fish liver tissue [18,34].

Group-wise survival and weight gain performance across all orthogonal treatments are summarized in Supplementary Table S2.

Histological scores were subjected to stratified one-way ANOVA within each temperature level, followed by Tukey’s HSD post hoc tests for within-temperature salinity comparisons. A global two-way ANOVA including temperature, salinity and their interaction was also performed but yielded no significant effects across the full dataset. All statistical analyses of histological scores were performed using the same ANOVA framework described in Section 2.10. It is noted that lesion scores represent ordinal rank data; parametric ANOVA was used here for exploratory descriptive comparison only, and these outputs should not be over-interpreted as definitive inferential evidence.

### 3.2. Overview of Hepatic Transcriptome Sequencing Quality

A total of 27 liver transcript libraries corresponding to nine orthogonal culture treatments were sequenced on the Illumina NovaSeq platform. After strict filtering of raw sequencing reads, all samples met the data quality standards for subsequent bioinformatic analysis (Supplementary Table S1), where detailed statistics including the Q20, Q30, GC content and genome mapping rate of each library were listed. The sequencing error rate of all samples was controlled below 0.013%, Q20 values ranged from 98.86% to 99.29%, and Q30 values were maintained at 94.46–96.62%. The GC content of each library remained stable between 45.36% and 49.00%, and the mapping rate of clean reads against the rainbow trout reference genome exceeded 95% for all samples.

**Figure 2:**
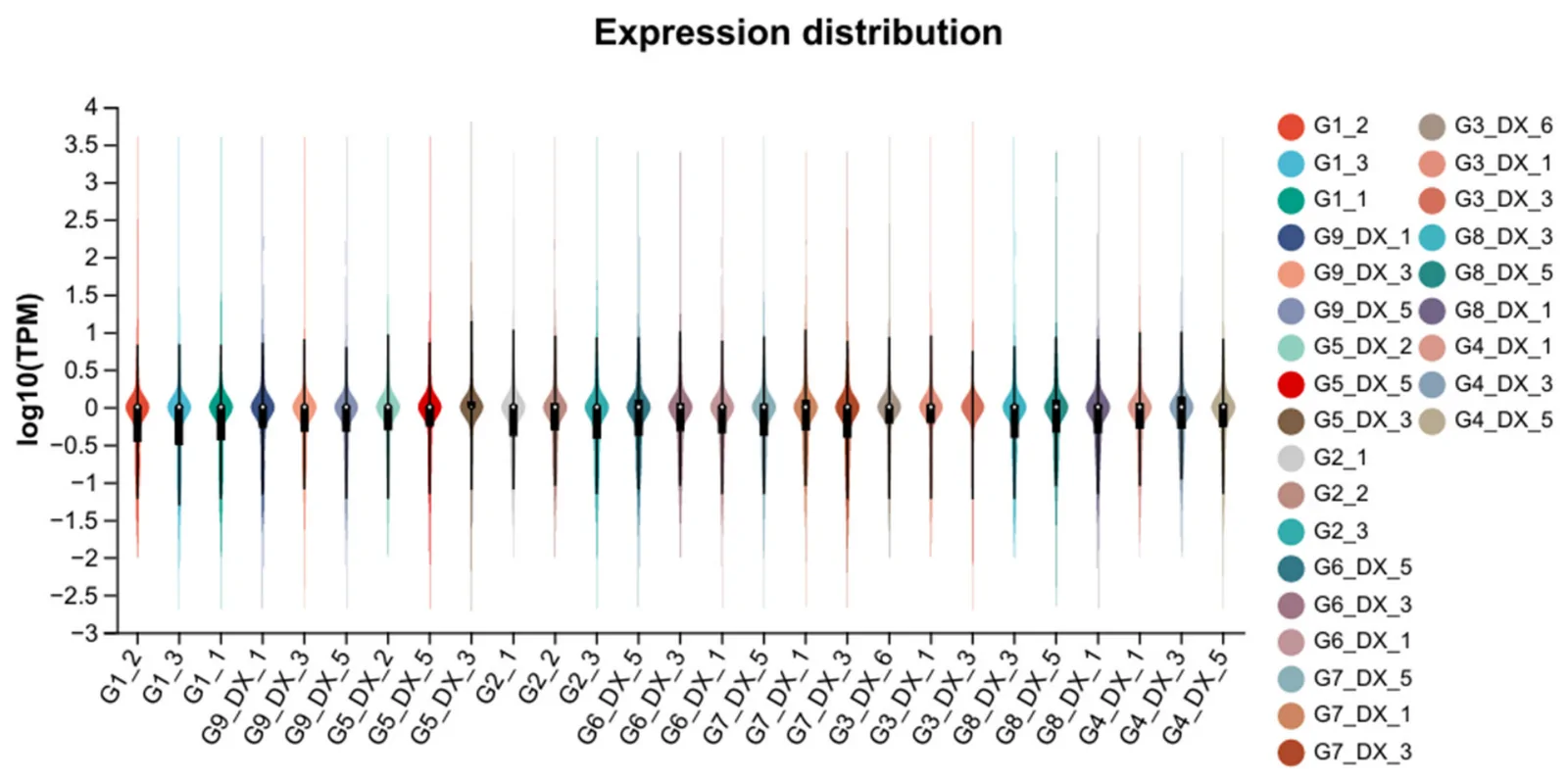
Global gene expression violin plot of all hepatic transcriptome samples, expression values converted to log10(TPM). Data are presented as mean ± SD (*n* = 3 biological replicates per group).

**Figure 3:**
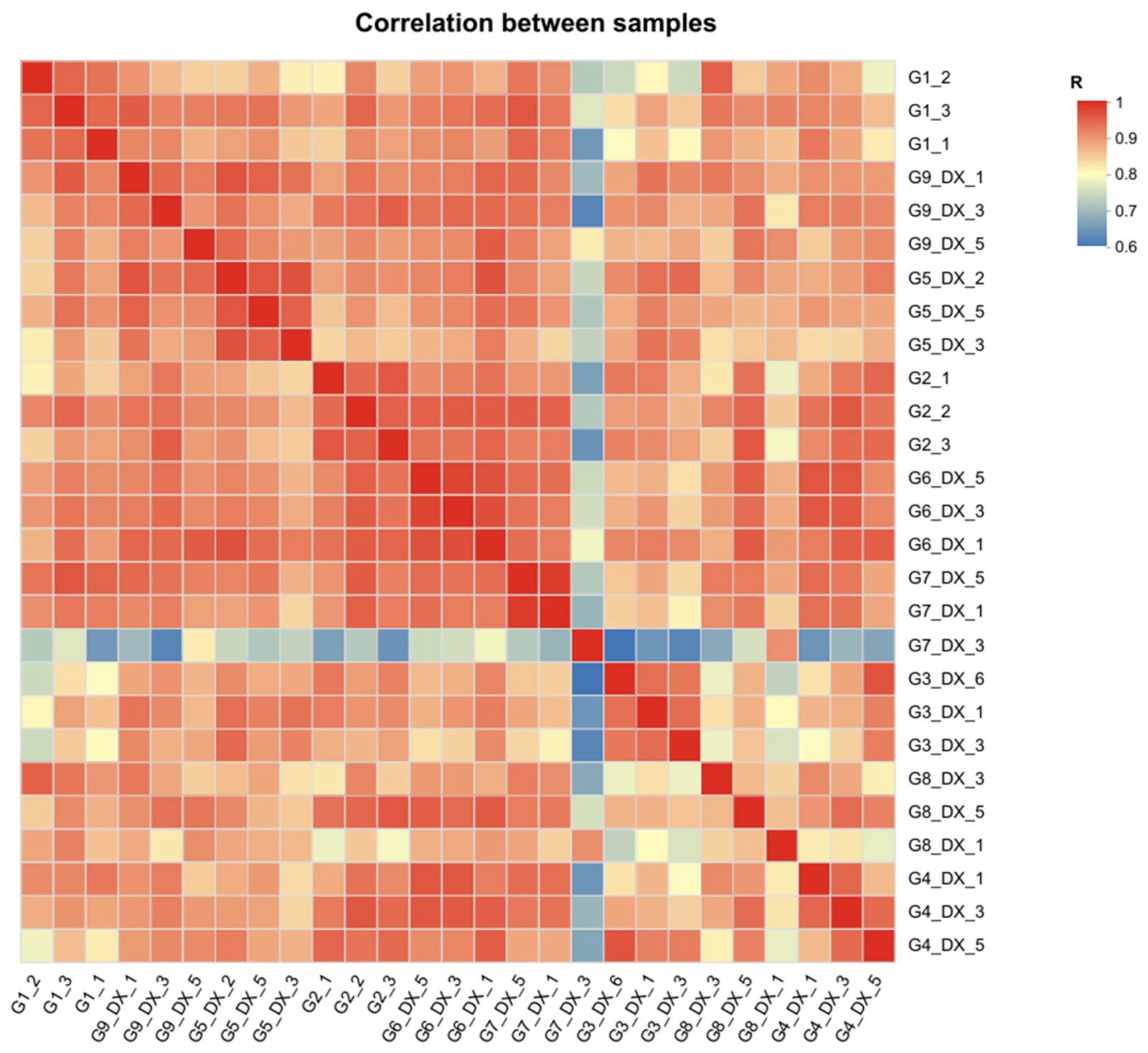
Pearson correlation heatmap of 27 liver biological replicates; color gradient represents correlation coefficient (blue = 0.6, red = 1.0). Data are presented as mean ± SD (*n* = 3 biological replicates per group).

The violin plot based on log10(TPM) values reflects the global gene expression distribution of all biological replicates (Figure 2). No obvious expression bias was observed across different culture groups, which indicates consistent RNA extraction, library construction and sequencing efficiency for all liver samples. The Pearson correlation heatmap of all samples further verified the high repeatability within each salinity main-effect subgroup; the correlation coefficients of biological replicates in the same salinity gradient were all higher than 0.9 (Figure 3).

**Figure 4:**
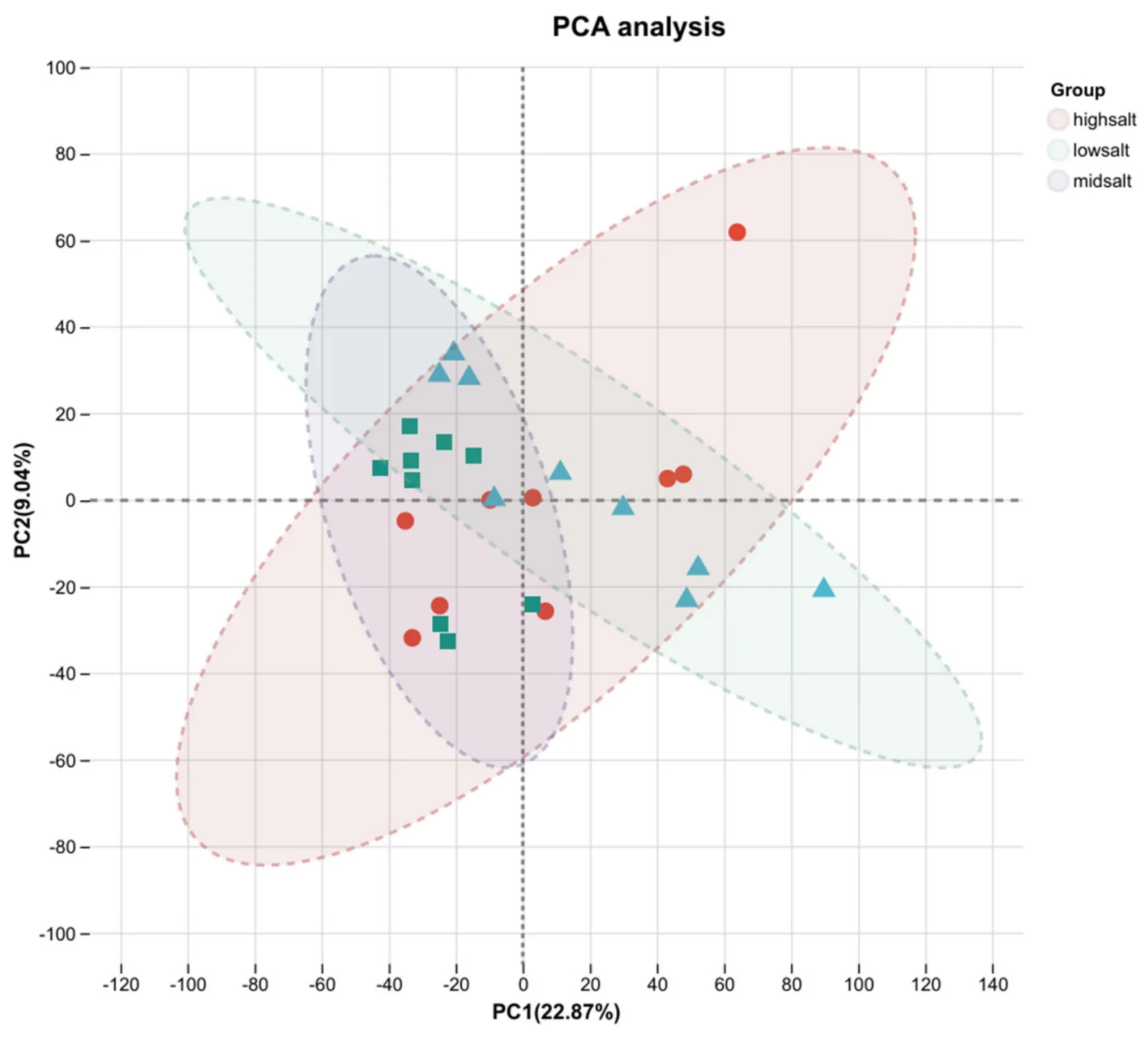
PCA plot grouped by salinity gradient. Light green ellipse = low salinity; light purple ellipse = medium salinity; light red ellipse = high salinity. Circles, squares and triangles represent samples from different body-weight groups. PC1 = 22.87%; PC2 = 9.04%. (*n* = 3 biological replicates per group).

Principal component analysis (PCA) was conducted to reveal transcriptional divergence among salinity main-effect groups (low salinity 10 ppt, medium salinity 20 ppt, and high salinity 30 ppt). PC1 explained 22.87% of the total transcriptional variation, and PC2 explained 9.04% (Figure 4). Samples of the three salinity gradients formed independent clustering ellipses, with a partial overlap between the low- and medium-salinity subgroups. The high-salinity group showed the most obvious transcriptional separation from the low-salinity control group, which indicates that sustained high salinity induced extensive genome-wide transcriptional remodeling in triploid rainbow trout liver tissue. Salinity was the primary source of transcriptional variation, while subtle sample shifts along the PC2 axis reflected temperature-induced transcriptional differences. Samples classified by body weight showed nearly overlapping distributions, which indicates that body weight imposes only mild regulatory effects on hepatic transcription under the present culture conditions.

### 3.3. Screening of Salinity-Responsive Differentially Expressed Genes (DEGs)

**Figure 5:**
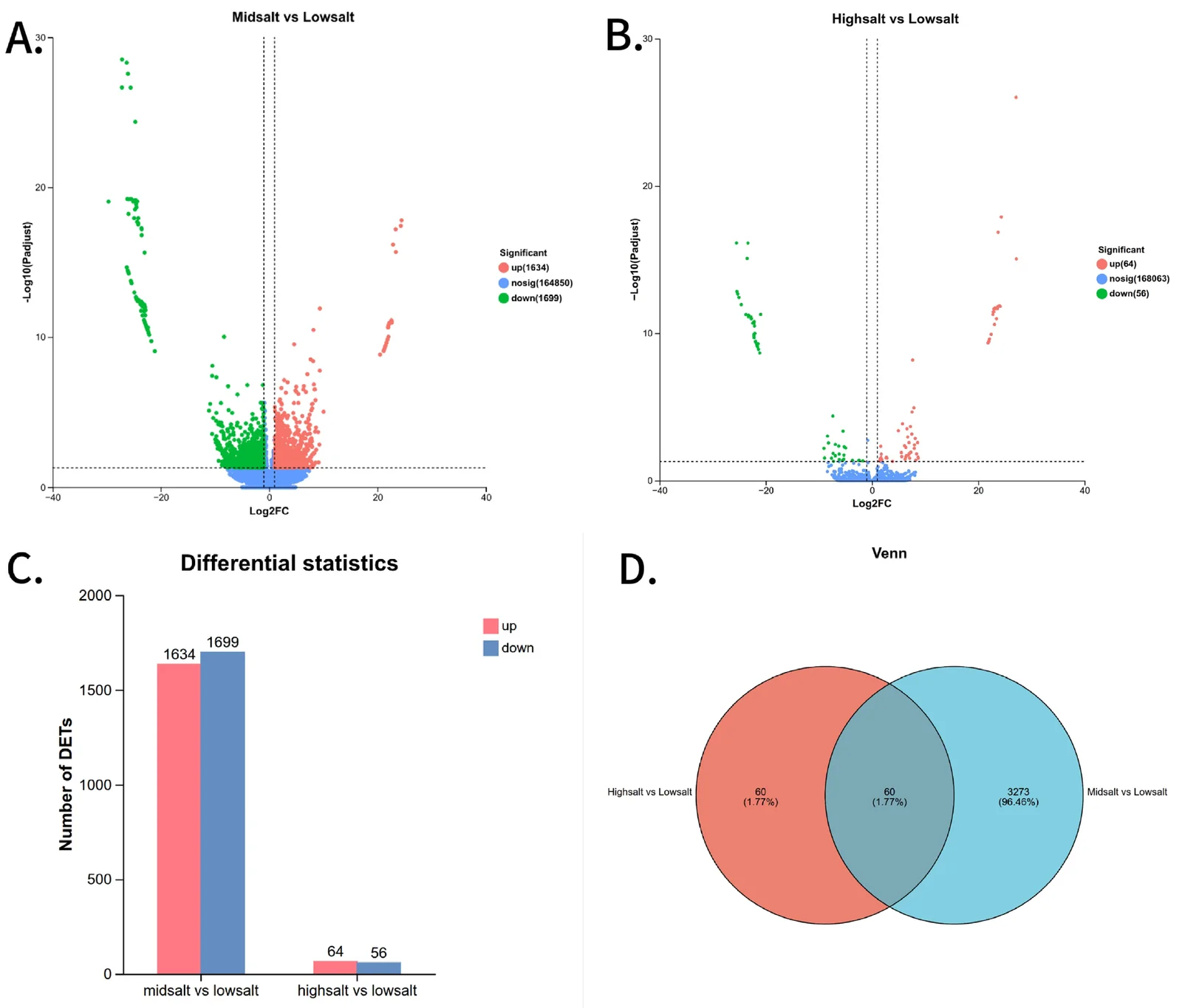
Summary of differentially expressed genes (DEGs) under two salinity comparisons. (**A**) Volcano plot, mid-salinity vs. low salinity; red = upregulated DEGs, green = downregulated DEGs, grey = non-significant genes. (**B**) Volcano plot, high salinity vs. low salinity; threshold |log_2_FC| ≥ 1, FDR < 0.05. (**C**) Bar chart showing counts of upregulated/downregulated DEGs in two comparison groups. (**D**) Venn diagram illustrating shared and unique DEGs between mid-salinity vs. low salinity and high salinity vs. low salinity. Data are presented as mean ± SD (*n* = 3 biological replicates per group).

Differentially expressed genes (DEGs) were screened using the DESeq2 R package with thresholds of |log_2_FC| ≥ 1 and FDR < 0.05. Instead of pair-wise comparisons between individual orthogonal tanks, we grouped samples by salinity main effects to eliminate confounding interference from body weight and temperature. All DEG visualization and statistical results are integrated in Figure 5.

Two salinity contrast groups were analyzed:

1. Medium salinity versus low salinity (Mid-S vs. Low-S): A total of 1634 significantly upregulated and 1699 downregulated DEGs were detected.

2. High salinity versus low salinity (High-S vs. Low-S): Only 64 upregulated and 56 downregulated DEGs were identified, which indicates far weaker transcriptional perturbation under extreme high salinity.

Volcano plots (Figure 5A,B) clearly show widespread gene expression shifts in the medium-salinity group, whereas most genes remained transcriptionally stable under high salinity. The bar chart (Figure 5C) quantifies the total number of differentially expressed transcripts in both comparisons, and the Venn diagram (Figure 5D) reveals only 60 overlapping DEGs between the two contrast groups. Specifically, 3273 DEGs were uniquely induced by medium salinity, while merely 60 transcripts exclusively responded to high salinity. These results demonstrated that medium and high salinity triggered fundamentally distinct transcriptional regulatory programs in triploid rainbow trout liver. These distinct transcriptional responses to medium and high salinity prompted further GO and KEGG functional enrichment analysis, consistent with previous hepatic salinity stress transcriptome findings in salmonids [9,35].

### 3.4. GO and KEGG Functional Enrichment Analysis of DEGs

#### 3.4.1. Functional Enrichment of Medium-Salinity-Induced DEGs

**Figure 6:**
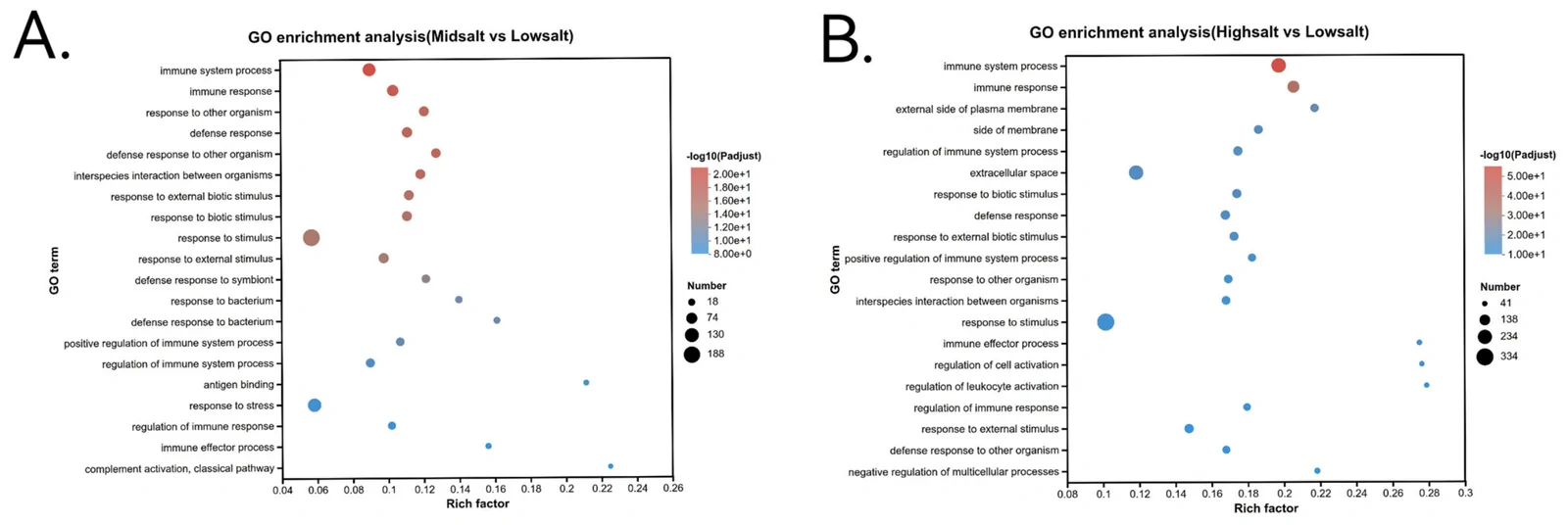
GO enrichment bubble plots for salinity-responsive DEGs (selected biological process terms). Bubble weight = enriched gene number, X-axis = Rich factor, color gradient = −log10(Padj). (**A**) Mid-salinity vs. low salinity; (**B**) high salinity vs. low salinity. Data are presented as mean ± SD (*n* = 3 biological replicates per group per comparison).

GO enrichment analysis (Figure 6A) demonstrated that the differentially expressed genes (DEGs) identified in the mid-salinity vs. low salinity comparison were predominantly enriched in biological processes related to immune defense and environmental stimulus response, including immune system processes, response to biotic stimulus, and complement activation. Significant enrichment was also detected in cellular component terms such as extracellular space and the plasma membrane outer region.

**Figure 7:**
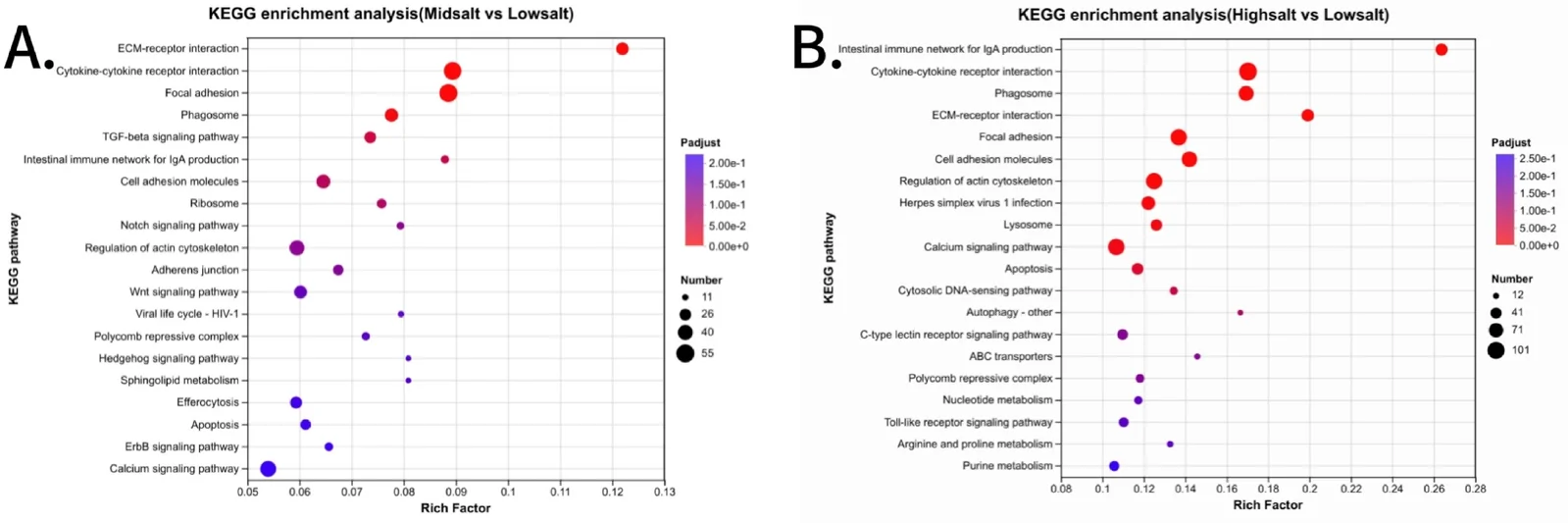
KEGG pathway enrichment bubble plots for salinity-responsive DEGs. Bubble weight = enriched gene number, X-axis = Rich factor, color gradient = −log10(Padj). (**A**) Mid-salinity vs. low-salinity; (**B**) high salinity vs. low salinity. Data are presented as mean ± SD (*n* = 3 biological replicates per group per comparison).

The corresponding KEGG enrichment results (Figure 7A) were dominated by immune signal transduction and cell adhesion pathways, with ECM–receptor interaction, cytokine–cytokine receptor interaction, focal adhesion and TGF-beta signaling ranking as the top enriched pathways. In addition, lipid metabolism (sphingolipid metabolism) and apoptotic signaling pathways were markedly activated in hepatic tissue under medium-salinity acclimation.

#### 3.4.2. Functional Enrichment of High Salinity-Induced DEGs

For the high salinity vs. low salinity comparison group, GO enrichment profiles (Figure 6B) remained centered on immune and defense-related biological processes. In contrast to the medium-salinity treatment, DEGs from high-salinity groups displayed stronger enrichment in functional terms associated with autophagy, apoptosis and oxidative stress.

KEGG pathway enrichment (Figure 7B) indicated that hyperosmotic stress significantly activated intestinal immune network, phagosome, apoptosis and calcium signaling pathways. Multiple energy metabolic cascades, including arginine and proline metabolism and purine and nucleotide metabolism, were drastically perturbed, triggering transcriptional perturbation of multiple energy metabolic cascades, which suggests a potential disturbance of hepatic energy homeostasis under high-salinity challenges.

Collectively, both medium- and high-salinity gradients triggered the core hepatic immune defense response. Medium salinity mainly altered pathways governing cell proliferation and cell adhesion; by contrast, extreme high salinity aggravated hepatocyte apoptosis and suggests the potential perturbation of systemic energy-related pathways at the transcript level [24]. Given that the above transcriptional changes reflected only the steady-state mRNA level, we therefore investigated whether salinity also modulated gene expression at the post-transcriptional level via alternative splicing.

### 3.5. Genome-Wide Alternative Splicing (AS and DAS) Profiles Along Salinity Gradients

**Figure 8:**
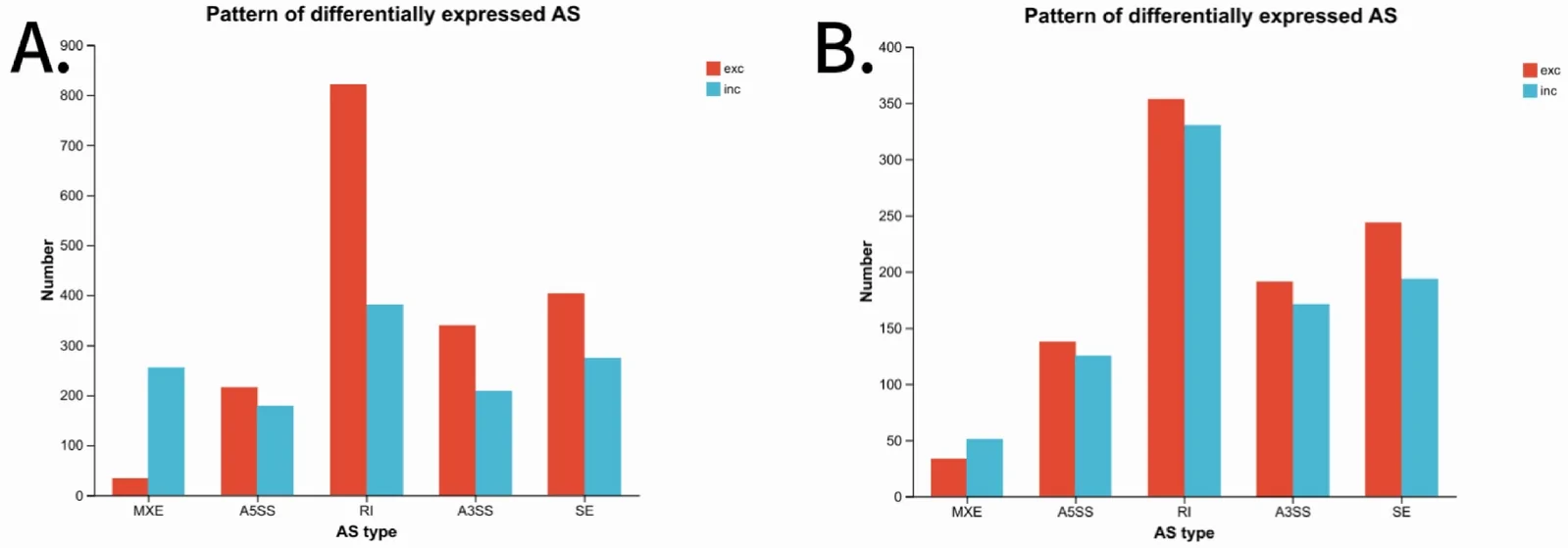
Classification statistics of all AS events. Red bars = excluded isoforms, blue bars = included isoforms. X-axis: MXE, A5SS, IR, A3SS, and SE. (**A**) AS event distribution, mid-salinity vs. low salinity group. (**B**) AS event distribution, high salinity vs. low salinity group. Data are presented as mean ± SD (*n* = 3 biological replicates per group).

rMATS software was applied to quantify five canonical alternative splicing (AS) subtypes (SE, IR, A5SS, A3SS, and MXE) in the mid-salinity and high salinity comparison group (Figure 8A,B). Intron retention (IR) was the most frequent AS event across both groups, followed by exon skipping (SE), alternative 3′ splice site (A3SS), alternative 5′ splice site (A5SS), and mutually exclusive exon (MXE). The total count of AS events identified under the mid-salinity treatment was markedly higher than that under high salinity, which matched the variation tendency of differentially expressed genes (DEGs). Mid-salinity exposure induced massive differential alternative splicing (DAS) events, which demonstrates that salinity fluctuation alone can extensively remodel hepatic post-transcriptional splicing landscapes under fixed temperature and body weight conditions. Temperature exerted moderate modulatory effects on DAS associated with apoptosis and endoplasmic reticulum stress at identical salinity gradients, whereas body weight barely triggered genome-wide splicing shifts. Functional enrichment of DAS-related genes revealed that splicing variants primarily participated in RNA processing, immune response and cellular stress signal transduction. This widespread post-transcriptional reprogramming aligns with previous teleost liver AS reports under osmotic stress [18,20]. The above statistics indicate that medium salinity induced far more genome-wide splicing alterations than the low- or high-salinity groups. To identify the core gene sets coordinating salinity and temperature responses, WGCNA was performed on all hepatic transcriptome data.

### 3.6. Weighted Gene Co-Expression Network Analysis

WGCNA was performed on the transcriptome data of 27 liver samples to identify gene modules associated with three experimental factors: body weight, temperature and salinity.

**Figure 9:**
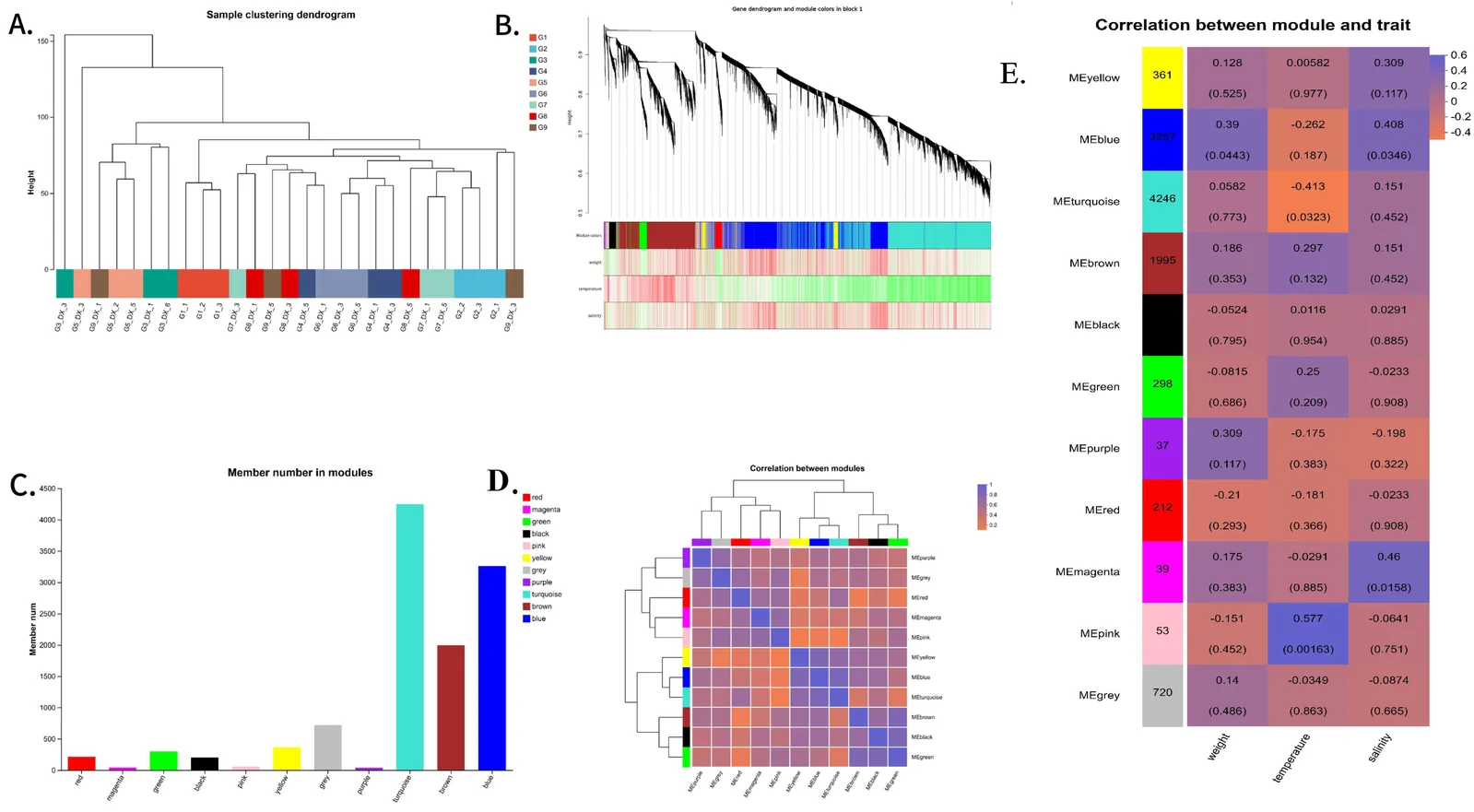
Hepatic transcriptome WGCNA under three-factor stress. (**A**–**E**) Sample tree, gene dendrogram, module gene counts, eigengene correlation and module–trait heatmaps with Pearson r and *p* values; significant correlations (*p* < 0.05) marked. Data are presented as mean ± SD (*n* = 3 biological replicates per group).

First, hierarchical clustering of all samples was performed to evaluate transcriptional similarity and exclude outliers (Figure 9A). No severely deviated individuals were detected, which proved the stable repeatability of the biological replicates within each orthogonal group. Subsequently, all expressed genes were grouped into distinct co-expression modules by dynamic tree cutting. The gene dendrogram matched with the module color bands and expression tracks of the three phenotypic traits is displayed in Figure 9B, and a total of 11 unique gene modules were obtained after filtering. The number of genes distributed in each module is quantified in the bar chart (Figure 9C); the turquoise module contained the maximum number of genes (4246), followed by the blue module (3257), while the purple and magenta modules had the fewest gene members.

The eigengene correlation heatmap was plotted to reveal the expression relevance between different modules (Figure 9D). Most modules maintained a moderate correlation, which indicates that each module undertakes relatively independent biological functions. The core module–trait correlation heatmap further screened gene sets significantly responding to temperature and salinity stress (Figure 9E).

The blue module was significantly positively correlated with salinity (*r* = 0.408, *p* = 0.0346) and identified as the key module responding to hyperosmotic stress, consistent with published WGCNA results regarding salmonid hepatic salinity response [18,33]. The pink module displayed a strong positive correlation with temperature (*r* = 0.577, *p* = 0.00163), while the turquoise module was negatively associated with temperature (*r* = −0.413, *p* = 0.0323). No module showed a statistically significant correlation with body weight (all *p* > 0.05), consistent with mild inter-group histological differences [20].

## 4. Discussion

### 4.1. Main Effect of Salinity on Rainbow Trout Survival and Hepatic Damage

Consistent with osmoregulatory research on teleost species [9,36], salinity was identified as the core limiting factor affecting triploid trout survival. Hyperosmotic environments disrupt systemic ion balance and force the liver to consume extra energy for osmotic adjustment, eventually triggering widespread oxidative hepatocyte injury. Consistent with salmonid stress research [9,37], only salinity exerted statistically significant independent impacts on survival, while temperature and body weight had no standalone significant effects on growth metrics, even though heat exposure could exacerbate hepatic lesions. Though body weight induced visible physiological differences, its independent regulatory capacity remained weak within the test gradient, consistent with previous observations that body size modulates osmoregulatory capacity through altered gill surface-area-to-volume ratios and metabolic scaling [38]. The superior stress resistance of large-bodied triploid individuals under low-temperature and low-salinity regimes reflects distinct endocrine and hepatic developmental programs compared with diploid conspecifics [35,37,39]. The 500 g + low temperature + low salinity combination delivers optimal stress tolerance, which provides direct grading guidance for offshore trout domestication [2,22].

### 4.2. Synergistic Interaction Between Temperature and Salinity on Liver Adaptive Response

Numerous teleost studies have documented that elevated temperatures can exacerbate salinity-induced physiological disturbances, including oxidative stress and tissue damage [4,5]. Concurrent thermal and hyperosmotic stress triggers severe hepatic structural destruction via overactivated oxidative and apoptotic cascades [21], which explains the sharp decline in survival under the combined high-temperature–high-salinity culture. Elevated basal metabolism under warm conditions accelerates ROS accumulation; when superimposed with salinity stimulation, ER stress and apoptotic pathways are overactivated, as demonstrated in the ER stress–oxidative stress interplay model [40], producing irreversible tissue lesions. Isolated mild salinity or low-temperature stimulation only triggers transient transcriptional adaptation and fails to produce irreversible liver lesions, consistent with previous flounder and salmonid reports [10,15]. This synergistic interaction highlights the necessity of precise temperature control during brackish domestication to avoid mass mortality [3].

### 4.3. Transcriptomic and Histological Synergistic Evidence of Hepatic Multi-Stress Adaptation

Histopathological observation supplies intuitive tissue-level proof of salinity-dominated liver injury, while RNA-seq uncovers the underlying molecular regulatory network [14,18]. Salinity acts as the primary driver reshaping hepatic metabolic and antioxidant transcriptional programs to maintain osmotic homeostasis [15,19]. Abundant differential alternative splicing (DAS) events are enriched in stress response transcripts, which verifies splicing as a critical post-transcriptional compensatory mechanism for salinity acclimation [17,32,33]. Medium salinity induces the most robust post-transcriptional remodeling via alternative splicing, whereas temperature only produces weak auxiliary regulation and body weight imposes negligible splicing shifts [18]. Such splicing patterns align with the salinity-correlated blue gene module screened via WGCNA [18,33]. WGCNA analysis identified the blue module as the core regulatory gene set responding to hyperosmotic stimuli, with temperature-related transcripts clustered within the pink module; no modules correlated significantly with body weight, which further validates the three-factor regulatory hierarchy [20,41,42]. Thermal stress universally amplifies salinity-triggered hepatic injury independent of fish developmental stage, a phenomenon also documented in Atlantic salmon [43]. Joint histological and multi-omics evidence collectively clarifies the independent and interactive regulatory logic of salinity, temperature and body weight in triploid trout liver.

### 4.4. Research Innovation, Production Guidance and Research Limitations

Most existing rainbow trout salinity trials only adopt single- or dual-factor experimental frameworks and ignore body weight as a research variable [11,35]. Unlike previous single-/two-factor trials, the L_9_ orthogonal framework in this work simultaneously evaluates three-factor main effects and explores potential correlative patterns among factors [2]. Combining HE histology and transcriptome and alternative splicing profiling, this paper constructs an integrated tissue molecular analytical system to fill research gaps on triploid trout multi-stress hepatic adaptation [12,31]. For offshore cage breeding, large-bodied triploids are recommended for domestication under low temperature and low salinity to maximize survival, while juveniles can be cultured at moderate salinity for faster growth; these findings suggest differentiated breeding schemes for China’s brackish aquaculture [2,43].

This research still has several limitations. The 60-day domestication cycle, while sufficient for detecting acute transcriptional and histological responses, cannot capture long-term adaptive changes across multiple growth cycles; future studies extending the acclimation period would complement our short-term findings [42]. Besides liver tissues, gill and intestinal tissues need supplementary sampling to build a complete whole-body osmoregulatory network [7,9]. The hub genes identified by WGCNA still require qPCR and hepatocyte in vitro functional verification; subsequent functional assays will be essential to confirm their roles in salinity tolerance [21]. Follow-up orthogonal trials can add dissolved oxygen gradients and time-series sampling to reveal dynamic short-/long-term hepatic adaptation trajectories [3]. Additionally, the skeletal abnormalities occasionally observed in triploid stocks under nutritional stress [44] warrant further investigation to assess their potential impact on overall fitness and seawater acclimation success.

Several inherent limitations should be acknowledged when interpreting our findings. First, the L_9_ (3^3^) orthogonal array design is optimized for evaluating main-factor effects; effect aliasing prevents robust, independent estimation for all pair-wise two-way interactions. Observed interactive-like patterns represent correlative trends inferred from histology and multi-omics datasets rather than statistically proven causal interactions. Full-factorial experimental designs are required in future work to formally test two-factor interactive effects. Second, our interpretation of energy homeostasis disturbance relies solely on transcript-level enrichment results, without direct metabolite, ATP or enzyme-activity measurements. Third, plasma osmolality, cortisol and feed conversion ratio were not quantified in this trial, which limits deeper physiological interpretation. A uniform 24 h fasting was applied for fish of all body weight classes; longer fasting would be preferable for gut clearance in large-sized individuals. In addition, this 60-day chronic trial captures mid-term stress responses, while long-term adaptive changes across multiple growth cycles remain unexplored. Further multi-tissue sampling, targeted metabolic assays and time-series experiments will enrich our understanding of triploid rainbow trout seawater acclimation.

## 5. Conclusions

Salinity was the only factor significantly affecting the survival of triploid rainbow trout, while temperature and body weight had no independent significant impacts on weight gain. High temperature was correlated with a greater severity of salinity-induced hepatic lesions under the tested orthogonal conditions, and the combination of 500 g, 10 °C and 10 ppt achieved optimal survival. Multi-omics and histology revealed that salinity remodeled hepatic transcription and alternative splicing to regulate osmoregulation, with temperature acting as a damage amplifier and body weight exerting trivial effects. Collectively, our integrated dataset reveals a suggestive three-factor regulatory hierarchy-like pattern under the tested experimental conditions and offers theoretical support for the size-specific seawater domestication of triploid rainbow trout [2,3].

## Figures and Tables

**Table 1:** Levels of three experimental factors.

Level	A: Body Weight (g)	B: Temperature (°C)	C: Salinity (ppt)
1	100	10	10
2	250	15	20
3	500	20	30

**Table 2:** Orthogonal experimental grouping scheme.

Treatment No.	A (Body Weight)	B (Temperature)	C (Salinity)	Culture Combination
1	1	1	1	100 g-10 °C-10 ppt
2	1	2	2	100 g-15 °C-20 ppt
3	1	3	3	100 g-20 °C-30 ppt
4	2	2	3	250 g-15 °C-30 ppt
5	2	3	1	250 g-20 °C-10 ppt
6	2	1	2	250 g-10 °C-20 ppt
7	3	3	2	500 g-20 °C-20 ppt
8	3	1	3	500 g-10 °C-30 ppt
9	3	2	1	500 g-15 °C-10 ppt

## Data Availability

The raw RNA sequencing data generated in this study have been deposited in the NCBI Sequence Read Archive (SRA) under BioProject accession number PRJNA1478201, which will be publicly accessible upon publication of this manuscript. All processed analysis data, Supplementary Tables and Figures are available within this article and its Supplementary Materials.

## Supplementary Materials

The following supporting information can be downloaded at: https://www.mdpi.com/article/10.3390/biology15181646/s1, Table S1. Summary of transcriptome sequencing quality metrics for 27 triploid rainbow trout liver samples, including sequencing error rate, Q20, Q30, GC content, raw reads, clean reads and clean base statistics. Table S2. Survival rate and weight gain rate of triploid rainbow trout under nine orthogonal experimental treatments after the 60-day chronic stress trial.
