# Supplementary material for: Combined Transcriptomic and Histological Profiling Uncover Hepatic Regulatory Hierarchy of Triploid Oncorhynchus mykiss Under Interactive Salinity, Temperature and Body Weight Regimes
Source: Biology (Basel). 2026 Sep 17;15(18):1646. doi: 10.3390/biology15181646 (PMC13604319; doi:10.3390/biology15181646)
Supplement: Supplementary file 1 [file biology-15-01646-s001.zip › biology-4479616-supplementary.pdf]

Table S1. Summary of RNA-seq quality metrics for 27 hepatic transcriptome libraries of triploid rainbow trout

Metric	Value Range
Sequencing Error Rate	0.0117% – 0.0126%
Q20	98.86% – 99.29%
Q30	94.46% – 96.62%
GC Content	45.36% – 49.00%
Raw Reads	28,480,616 – 48,487,622
Clean Reads	26,664,662 – 47,202,022
Clean Bases	6.31 Gb – 9.64 Gb

Table S2. Survival rate and weight-gain rate of triploid rainbow trout under nine orthogonal experimental treatments after the 60-day chronic stress trial

Treatment ID	Treatment combination (Body weight-Temperature-Salinity)	Survival rate (%)	Weight-gain rate (%)
1	100 g-10 °C-10 ppt	96.7	32.7
2	100 g-15 °C-20 ppt	85.5	43.5
3	100 g-20 °C-30 ppt	18.2	38.2
4	250 g-15 °C-30 ppt	44.3	5.2
5	250 g-20 °C-10 ppt	55.6	10.4
6	250 g-10 °C-20 ppt	97.1	15.1
7	500 g-20 °C-20 ppt	65.1	3
8	500 g-10 °C-30 ppt	36.4	11
9	500 g-15 °C-10 ppt	100	9.2

Note: Each treatment contained two replicate tanks; values represent overall pooled performance for each treatment group. Formal two-way interaction terms cannot be statistically tested with this L_9 orthogonal layout.
